# Exposure to Bat-Associated *Bartonella* spp. among Humans and Other Animals, Ghana

**DOI:** 10.3201/eid2205.151908

**Published:** 2016-05

**Authors:** Alexandra O. Mannerings, Lynn M. Osikowicz, Olivier Restif, Edward Nyarko, Richard Suu-Ire, Andrew A. Cunningham, James L.N. Wood, Michael Y. Kosoy

**Affiliations:** University of Cambridge, Cambridge, UK (A.O. Mannerings, O. Restif, J.L.N. Wood);; Zoological Society of London, London, UK (A.O. Mannerings, A.A. Cunningham);; Centers for Disease Control and Prevention, Fort Collins, Colorado, USA (L.M. Osikowicz, M.Y. Kosoy);; Public Health Division, 37 Military Hospital, Accra, Ghana (E. Nyarko);; Forestry Commission, Accra (R. Suu-Ire).

**Keywords:** Bartonella spp., zoonoses, fruit bats, disease spillover, Ghana, bacteria

**To the Editor:** Human contact with wildlife is a leading cause of disease spillover. Bats, in particular, host numerous zoonotic pathogens, from henipaviruses to lyssaviruses (*1*). In Ghana, the straw-colored fruit bat (*Eidolon helvum*) frequently and closely interacts with humans through roosting in urban areas and human harvesting of bushmeat. Large colonies live in Accra, the capital city, and >128,000 bats, on average, are hunted for food yearly in southern Ghana alone (*2*). Serologic evidence of human infection with novel paramyxoviruses from *E. helvum* bats (*3*) supports concerns regarding this contact. In addition, Kosoy et al. (*4*) isolated several new strains of *Bartonella* that were found in >30% of *E. helvum* bats, whereas Billeter et al. found *Bartonella* in 66% of their ectoparasites (*5*), with *Bartonella* transmissibility to other species unknown. This prevalence causes concern because many *Bartonella* species are zoonotic and cause substantial human disease (*6*). Previous studies of febrile patients in Thailand have shown prevalence rates of <25% for antibodies against zoonotic *Bartonella* species (*7*). Serologic studies have been conducted in Europe and in the United States, but few studies have examined such prevalence in Africa among patients and in the general population (*8*). 

To address these concerns, we conducted a prevalence study in Ghana, West Africa, for evidence of bat-associated *Bartonella* infection in humans and other common animal species. We sampled humans who had close contact with fruit bats and also sampled domestic animals that lived around the bat colonies.

We obtained serum samples from 335 volunteers from Accra and the Volta region who had close contact with *E. helvum* bats and also sampled 70 domestic animals that lived underneath bat colonies (5 cats, 23 chickens, 7 cows, 6 dogs, 21 goats, 8 sheep) in Accra. We used 3 testing approaches: culture, PCR, and indirect immunofluorescent assays for serologic testing. We tested serum specimens for antibodies against *B. henselae*, *B. quintana*, *B. clarridgeiae*, *B. vinsonii vinsonii*, *B. elizabethae*, and *Bartonella* strain E1–105, which had been isolated from *E. helvum* bats (*6*).

All culture results for human and domestic animal samples were negative for *Bartonella* species. One human serum sample was positive for *B. clarridgeiae* by PCR, which was confirmed by repeat testing. No other human samples were consistently positive by PCR. Of 70 animal blood clots and 62 serum samples tested by using PCR, 1 serum sample from a cat tested positive for *B. henselae*. One human serum sample was positive by immunofluorescent assay for antibodies against *B. henselae* at titers of 1:128, another had reactivity to *B. henselae* at 1:64, and 1 sample was reactive at 1:32. Five human serum samples were reactive to *B. quintana* at titers of 1:32.

The absence of evidence of any human exposure to bat-associated *Bartonella* suggests that the species isolated from *E. helvum* bats never or rarely infects humans in Ghana. If *Nycteribiidae* bat flies serve as the vector for *Bartonella* transmission between bats as hypothesized, then the high host specificity of these vectors (*8*) could explain why little infection is spilling over to other species. However, no experimental studies have confirmed that bat flies are competent vectors of bat-associated *Bartonella* species or that these ectoparasites only bite bats. These facts must be confirmed because bat flies are occasionally found on other animals and whether the parasites can successfully use these animals as hosts is unknown (*9*). Although further studies are needed to clarify the dynamics of *Bartonella* species infection in *E. helvum* bats, as well as the species’ zoonotic potential, the current risk of spillover of this bat-associated *Bartonella* species appears low in West Africa. This fact may be useful in directing limited public health resources.

The seroprevalence to *B. henselae* in healthy human participants in this study was <1%. The low levels of seropositivity to *B. henselae* and *B. quintana* are consistent with those found in the only other study on *Bartonella* in humans in sub-Saharan Africa: a survey of 155 subjects in the Democratic Republic of Congo showed 1% seroprevalence of *B. henselae* and <1% seroprevalence of *B. quintana* (*8*).

The results of study in the Democratic Republic of Congo and this study contrast with some studies in Asia and Europe, which show higher rates of human exposure to *Bartonella* species. For example, a study of febrile patients in Thailand found serologic evidence of exposure to *Bartonella* infection in 25% of patients (*7*).

Laudisoit et al. (*8*) were, to our knowledge, the first to report evidence of *Bartonella* infection in humans in Africa. Our study contributes to this nascent effort of understanding *Bartonella* on the continent. Because a substantial proportion of *Bartonella* prevalence studies have been done on hospital patients, our study provides a survey of the general population to help determine background infection rates and illuminate the complex risks posed by this zoonosis.
